# Supplementary material for: Influences of Crystallinity and Crosslinking Density on the Shape Recovery Force in Poly(ε-Caprolactone)-Based Shape-Memory Polymer Blends
Source: Polymers (Basel). 2022 Nov 4;14(21):4740. doi: 10.3390/polym14214740 (PMC9658307; doi:10.3390/polym14214740)
Supplement: Supplementary file 1 [file polymers-14-04740-s001.zip › polymers-1969720-supplementary.pdf]

Supplementary Information: Influences of Crystallinity and Crosslinking Density on the Shape Recovery Force in Poly(ε-Caprolactone)-Based Shape-Memory Polymer Blends

Ailifeire Fulati [1,2], Koichiro Uto [1] and Mitsuhiro Ebara [1,2,3,*]

1 Research Center for Functional Materials, National Institute for Materials Science, Tsukuba 3050044, Japan
2 Graduate School of Science and Technology, University of Tsukuba, Tsukuba 3058577, Japan
3 Graduate School of Advanced Engineering, Tokyo University of Science, Tokyo 1258585, Japan
* Correspondence: ebara.mitsuhiro@nims.go.jp

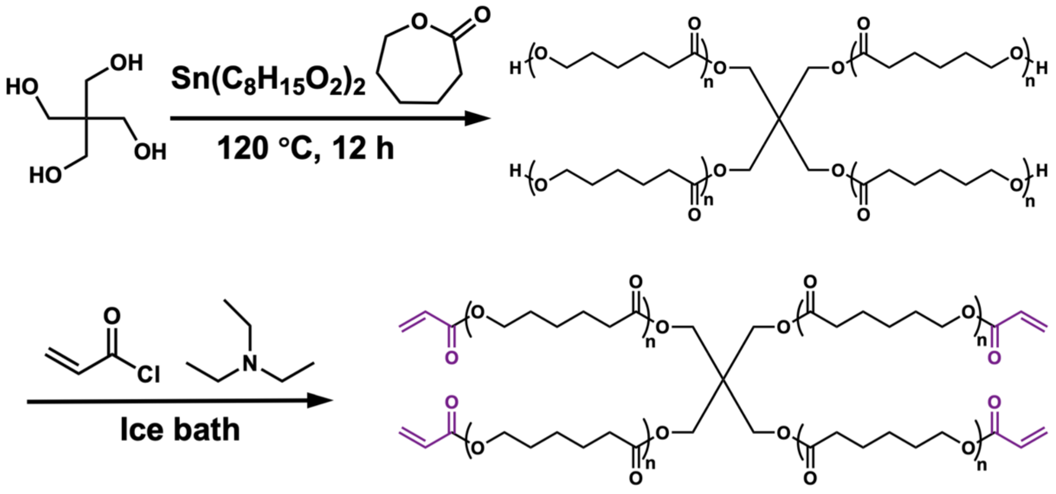

Scheme 1. Synthesis scheme of 4bPCL.

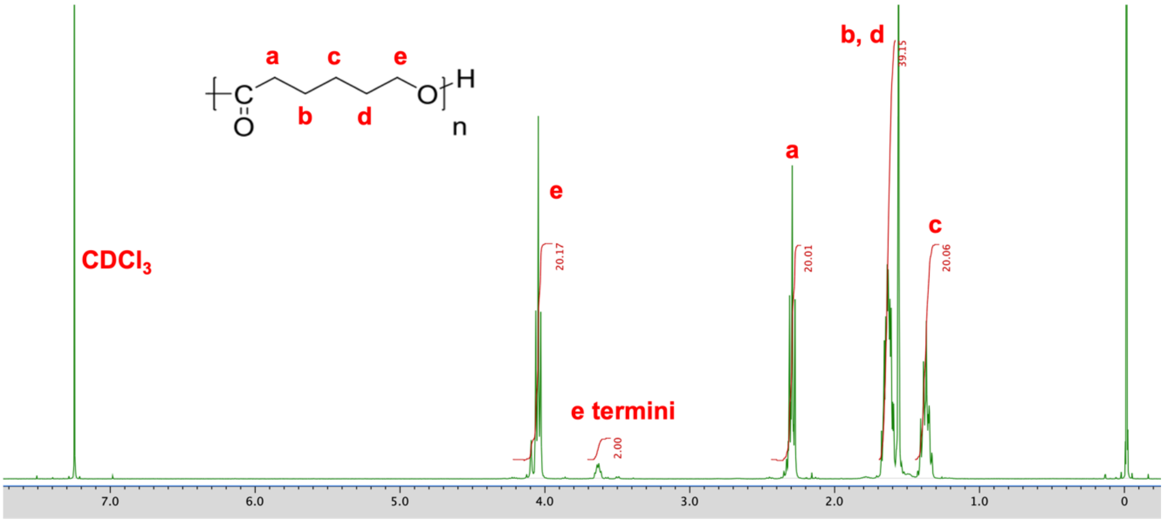

Figure S1. Representative ^{1}H-NMR spectra of 4b10 PCL

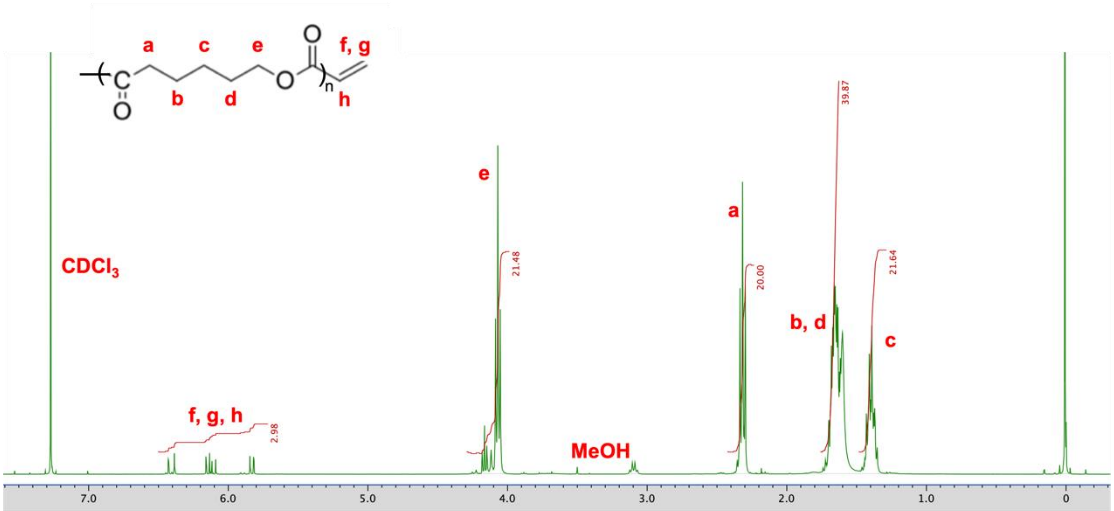

Figure S2. Representative ^{1}H-NMR spectra of 4b10 PCL macromonomer

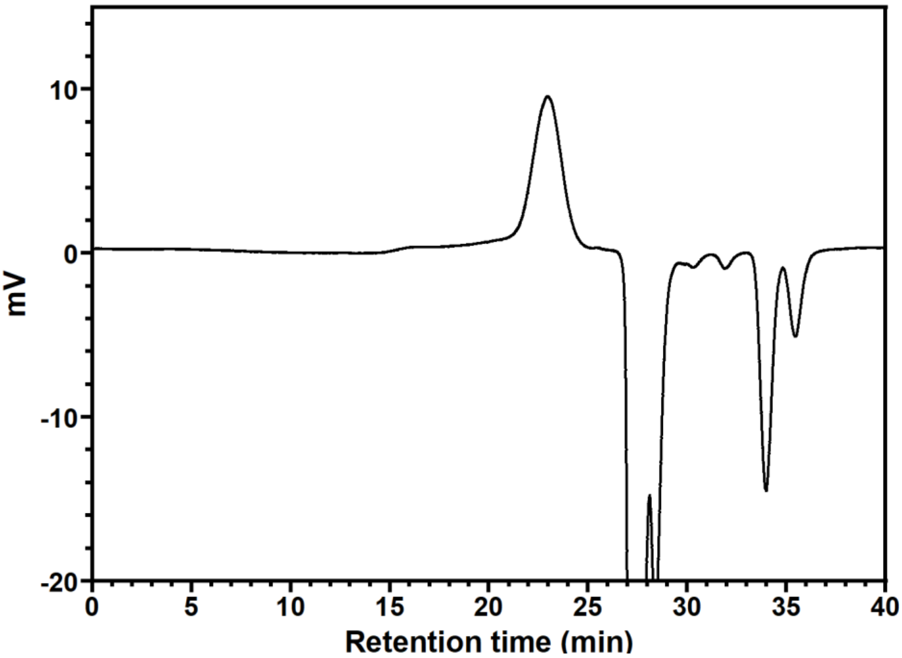

Figure S3. Representative GPC trace of 4b10PCL

Table S1. Molecular weights, polydispersity indexes and end group introduction rates of 4b10PCL and 4b100PCL macromonomers.

Polymer type	Mn (Da) [1)]	Mw (Da) [1)]	PDI [1)]	I.R. (%) [2)]
4b10 PCL-macro	6296	7022	1.115	99.3
4b100 PCL-macro	41126	63661	1.550	95.2

I.R.: End group introduction rate

1) Measured by GPC. Solvent: DMF. Standard: PS.

2) Calculated by ^{1}H NMR; solvent: $CDCl_3$

Table S2. Thermal properties of 4b100PCL/4b10PCL blend polymer systems.

**Sample**	**T_m (°C)**	**T_c (°C)**	**ΔH_m (mJ/mg)**	**ΔH_c**	**χ_c (%)**
**4b100PCL/4b10PCL_100:0**	57.3	27.7	53.6	-50.1	39.41
**4b100PCL/4b10PCL_90:10**	53.9	28.3	43.9	-51.1	32.28
**4b100PCL/4b10PCL_80:20**	53.2	28.5	43	-50.9	31.62
**4b100PCL/4b10PCL_70:30**	52.1	25.5	40.4	-50.4	29.71
**4b100PCL/4b10PCL_60:40**	51	24.3	37.3	-46.4	27.43
**4b100PCL/4b10PCL_50:50**	49.5	22.8	34.7	-42.9	25.52
**4b100PCL/4b10PCL_40:60**	46.6	15.4	28.1	-35.9	20.66
**4b100PCL/4b10PCL_30:70**	42.2	2.4	25	-28.6	18.38
**4b100PCL/4b10PCL_20:80**	38.1	1.8	20.9	-27.9	15.37
**4b100PCL/4b10PCL_10:90**	26.3	-10.9	18.4	-22	13.53
**4b100PCL/4b10PCL_0:100**	20.5	-19.1	14.5	-11	10.66

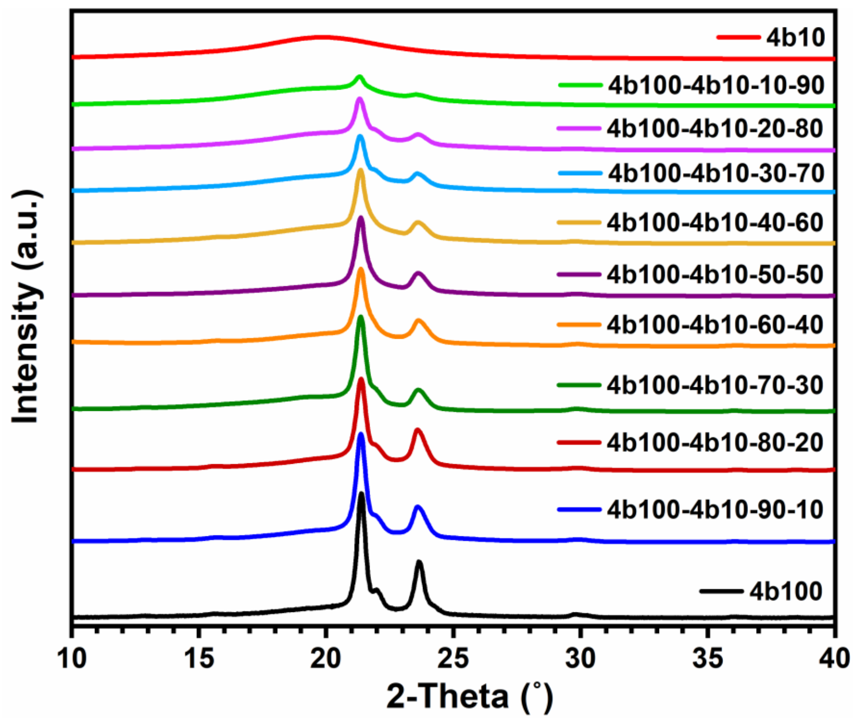

Figure S4. XRD patterns of 4b100PCL/4b10PCL blend polymer films.

Table S3. Mechanical properties of 4b100PCL/4b10PCL blend polymer systems in the uniaxial tensile test at room temperature.

Sample	Elastic modulus (MPa)	Toughness (MJ/m^3)	Strain at break (%)
**4b100PCL/4b10PCL_100:0**	250.4	130.4	1003.6
**4b100PCL/4b10PCL_90:10**	185.1	105.0	952.6
**4b100PCL/4b10PCL_80:20**	183.0	102.2	872.0
**4b100PCL/4b10PCL_70:30**	159.5	51.6	549.2
**4b100PCL/4b10PCL_60:40**	144.9	41.5	359.5
**4b100PCL/4b10PCL_50:50**	90.2	32.0	315.3
**4b100PCL/4b10PCL_40:60**	69.6	24.7	283.9
**4b100PCL/4b10PCL_30:70**	27.3	14.7	245.5
**4b100PCL/4b10PCL_20:80**	16.6	8.1	197.9
**4b100PCL/4b10PCL_10:90**	9.9	4.5	151.6
**4b100PCL/4b10PCL_0:100**	2.6	0.6	86.5

Table S4. Mechanical properties of 4b100PCL/4b10PCL blend polymer systems in the uniaxial tensile test at 60ºC.

Sample	Elastic modulus (MPa)	Stretchability (%)
**4b100PCL/4b10PCL_100:0**	0.5772	1419.5
**4b100PCL/4b10PCL_90:10**	0.6677	1049.0
**4b100PCL/4b10PCL_80:20**	0.7221	705.3
**4b100PCL/4b10PCL_70:30**	0.8579	576.1
**4b100PCL/4b10PCL_60:40**	0.8740	299.6
**4b100PCL/4b10PCL_50:50**	1.0302	156.3
**4b100PCL/4b10PCL_40:60**	1.2391	133.6
**4b100PCL/4b10PCL_30:70**	1.2766	110.0
**4b100PCL/4b10PCL_20:80**	1.6381	63.2
**4b100PCL/4b10PCL_10:90**	1.8530	57.5
**4b100PCL/4b10PCL_0:100**	2.0528	50.5
